# Investigating the Blink Reflex Abnormality in Dry Eye Patients

**DOI:** 10.7759/cureus.49741

**Published:** 2023-11-30

**Authors:** Uğur Yılmaz, Selma Tekin, Burak Elibol, Zehra Yalçındağ

**Affiliations:** 1 Ophthalmology Department, Pamukkale University, Denizli, TUR; 2 Neurology Department, Pamukkale University, Denizli, TUR

**Keywords:** trigeminal nerve, facial nerve palsy and sjogren, reflex, blinking, dry eye disorder

## Abstract

Introduction

Dry eye is an ocular surface disorder caused by increased evaporation, decreased tear production, or mixed form. Tears are secreted by the lacrimal gland after lacrimal nerve stimulation connected to the facial nerve. In nerve damage, tear secretion decreases, and dry eye develops. Our aim is to investigate the presence of pathology in the facial trigeminal nerve and neuronal pathways that provide reflex connections between these nerves by measuring the blink reflex in patients with dry eyes.

Methods

Schirmer test and tear breakup time were performed. Tear breakup time measurement was repeated three times, and the average of three was accepted. Tear breakup time <10 seconds and Schirmer test <10 mm without local anesthesia were accepted as dry eye. Patients having traumatic corneal pathology, ectatic corneal disease, inflammatory and microbial keratitis, previous ocular surgery, glaucoma, diabetic retinopathy, and chronic neurological diseases were excluded. The control group was randomly formed from 42 eyes of 21 healthy volunteers. Blink reflex was measured in both groups, and the R1 and R2 responses of the two groups were compared. Written consent was obtained from the patient (or legal guardian) so that her medical data could be published.

Results

There was no significant difference between the two groups in R1 and R2 responses in both eyes. There was no significant difference in terms of gender between the two groups (p=0.100). The mean age in the patient group was significantly higher than in the control group (p<0.000). The mean Schirmer test in the patient group was 8.6±1.1 mm in the right eye and 8.97±1.0 mm in the left eye.

Conclusion

There was no central pathology observed in terms of reflex blinking in dry eye disease. However, in future studies, brainstem fiesta sequence magnetic resonance imaging (MRI) can be planned to evaluate central pathologies in more detail.

## Introduction

Dry eye is an ocular surface disorder caused by increased evaporation, decreased tear production, or mixed form [1-3]. Topically lubricating with artificial tears, topical anti-inflammatory drops such as steroids and topical cyclosporine are used for its treatment. Tears are secreted by the lacrimal gland after lacrimal nerve stimulation connected to the facial nerve. In nerve damage, tear secretion decreases and dry eye develops. Inflammation and increased osmolarity because of dry eye can affect the corneal nerves, initiating a vicious cycle that causes somatosensorial abnormalities. Corneal sensitivity and reflex tear secretion are reduced by damage to the corneal nerves and dry eye severity increases. For this reason, recently, studies have been focused on neural mechanisms in dry eye, and drugs that increase tear secretion by neural mechanisms were evaluated [4,5].

Blinking helps tears spread across the surface of the eye. It also helps the glands' secretion in the eyelid prolonging the duration of tears on the ocular surface. Blink rate and interval are affected by ocular surface features [6]. Decreased and insufficient blinking causes increased ocular surface symptoms in dry eye patients at the end of the day [7]. There are studies investigating blink rate and inter-blink interval abnormalities in dry eye disease [6,8]. The afferent innervation of the blink reflex is provided by the supraorbital branches of the trigeminal nerve, and the efferent innervation is provided by the bilateral facial nerve. The blink reflex test consists of two responses, ipsilateral R1 and delayed bilateral R2. The R1 response is the disynaptic pontine reflex pathway between the trigeminal nerve main sensory nucleus and the ipsilateral facial nerve nucleus. The R2 response is a pathway from the spinal tract nucleus of the trigeminal nerve to the ipsilateral and contralateral facial nerve nuclei, including the pons and lateral medulla. These mechanisms can be impaired in many neurological diseases and facial and trigeminal nerve damage.

In this study, we aimed to investigate the presence of pathology in the facial trigeminal nerve and neuronal pathways that provide reflex connections between these nerves by measuring the blink reflex in patients with dry eyes.

## Materials and methods

This study includes 46 eyes of 23 dry eye patients diagnosed in Pamukkale University Medical School Training and Research Hospital. A detailed ophthalmological examination including vision, intraocular pressure (with noninvasive, noncontact tonometer), and anterior segment and retinal examination. Schirmer test and tear and breakup time were performed on all volunteers to investigate the presence of dry eye. First, a Schirmer test without local anesthesia was performed. After the Schirmer test, the tear breakup time measurement was repeated three times, and an average of three was accepted. A Schirmer test <10, and tear breakup time <10 seconds mm were accepted as dry eye. Patients having traumatic corneal pathology, ectatic corneal disease, contact lens usage, inflammatory and microbial keratitis, previous ocular surgery, glaucoma, diabetic retinopathy, and chronic neurological diseases with/without ocular muscle involvement like Bell’s Palsy, Parinaud Syndrome, cerebrovascular diseases, chronic neurodegenerative diseases like Alzheimer's disease, Parkinson's disease, prion disease, multiple sclerosis, amyotrophic lateral sclerosis, motor neuron disease, Huntington's disease, spinal muscular atrophy, and spinocerebellar ataxia were excluded. The control group was formed of 42 eyes of 21 healthy volunteers. Two groups were compared. Written consent was obtained from the volunteers (or legal guardians) so that their medical data could be published. This study was approved Institutional Review Board of Pamukkale University, School of Medicine. Approval date and number are 12/11/2020-68399.

Blink Reflex Measurement

**Figure 1:**
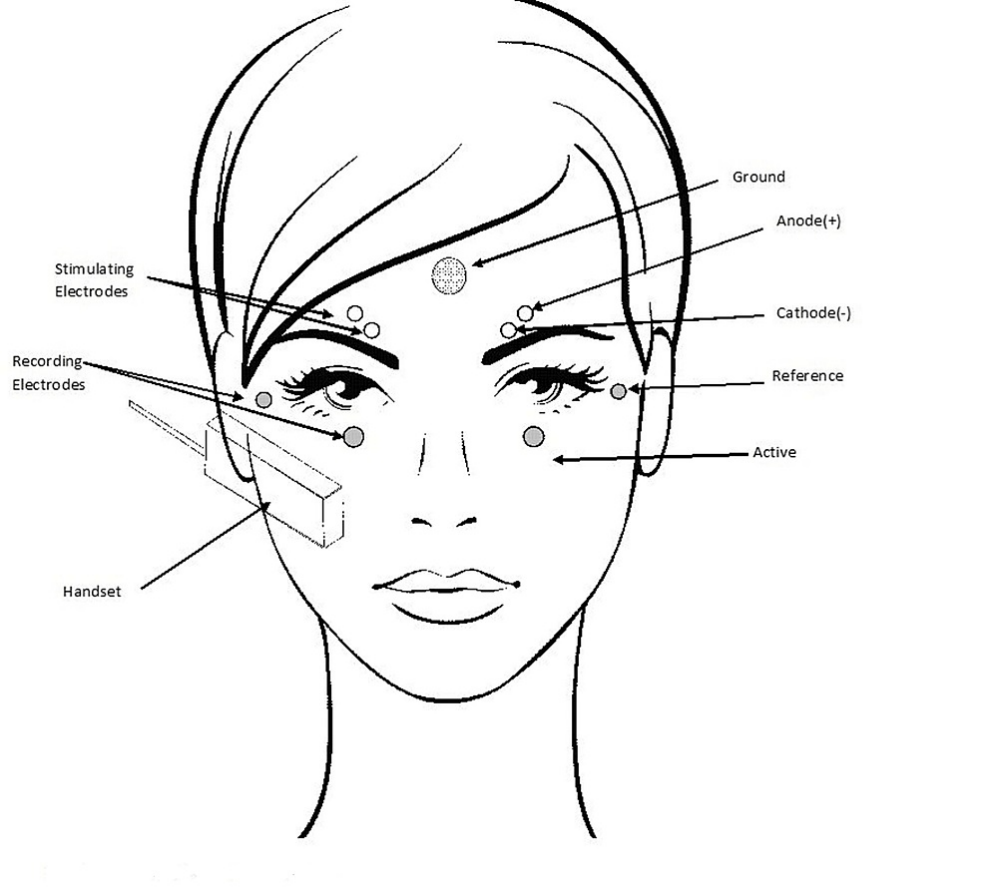
Blink reflex measurement The figure is the authors' own creation.

Blink reflex was studied by a Medelec Premier Plus (Medelec Woking, England) model electroneuromyography device. The blink reflex test was examined by the same person with the same method. For each Blink reflex test, the ground electrode is placed on the subject’s forehead, the active electrode is placed over both orbicularis oculi muscles, just below the eyes, and the reference electrode is placed on the outer canthus of both eyes (Figure 1). Stimulation is done by placing the cathode directly on the supraorbital branch of the trigeminal nerve, with the anode 3 cm above the cathode on the forehead. Following the dual simultaneous recording, four responses obtained from each side were superimposed, and one response was selected to define the shortest R1 and R2 delays (R1 is the delay of the deviation of the shortest initial recorded potentials. R2 is the delay of the second shortest potentials).

Statistical Analysis

Statistical Product and Service Solutions (SPSS) 21 software (IBM Corporation, Armonk, NY, USA) was used for statistical analyses. The independent samples T-test and Pearson’s correlation tests were used to compare the age and blink reflex measurements of the groups. Pearson's chi-square test was applied to compare two groups for gender. A value of p<0.05 was accepted as statistically significant.

## Results

There was no significant difference between the two groups in R1 responses in the right eyes (p=0.481, 0.664, respectively). R2 response was greater in the control group compared to the patient group, but the difference was not significant (p=0.664). There was no statistically significant difference between the patient and control groups in terms of R1 and R2 responses in the left eyes (p=0.778, 0.250, respectively) (Table 1).

**Table 1:** Blink reflex measurements of groups

	Patient Group (N=46) Mean±SD	Control Group (N=42) Mean±SD	P-values
Right R1 (ms)	11.1±1.6	10.5±1.2	0.481
Right R2 (ms)	33.2±4.6	35.1±14.8	0.664
Left R1 (ms)	10.7±1.1	10.5±1.0	0.778
Left R2 (ms)	31.5±7.5	31.5±3.8	0.250
p<0.05 is statistically significant; ms: millisecond			

There was no significant difference in terms of gender between the two groups (p=0.100). The mean age in the patient group was significantly higher than in the control group (p<0.0001) (Table 2).

But there was no correlation between age and R1 and R2 response of the right and left eyes (respectively, p=0.484, 0.639, 0.637, and 0.807).

No significant difference was observed between the two groups in terms of vision and intraocular pressure (p=0.778, 0.250, respectively) (Table 2). The mean Schirmer test in the patient group was 8.6±1.1 mm in the right eye and 8.97±1.0 mm in the left eye in the patient group.

**Table 2:** Demographic characteristics of the groups

		Patient Group (N=23)	Control Group (N=21)	P-values
Age (Mean±SD)		48.0±12.5	26.3±7.8	<0.001
Gender N(%)	Female	25 (86,2%)	14 (66,7%)	0.10
	Male	4 (13.8%)	7 (33.3)	
Vision (LogMAR)		0.00	0.00	1.00
Intraocular pressure (Mean±SD)		18±2.0	17±2.5	0.78
p<0.05 is statistically significant				

## Discussion

It is defined that ocular surface neurosensorial abnormalities play a key role in dry eye disease [9]. In their study, Tepelus et al. found decreased corneal nerve density and increased corneal inflammatory dendritic cells measured in dry eye patients by confocal microscopy. Moreover, they found a negative correlation between corneal nerve density, reflectivity, and ocular surface disease index score [10]. Increased osmolarity, mechanical stress, and chronic inflammation can lead to proinflammatory neuropeptide secretion, resulting in neuronal abnormalities [11]. The density of corneal nerves decreases because of many reasons such as diabetes mellitus and infectious keratitis. However, in studies, corneal nerve density decreased in some patients with dry eye [12], increased in some [13], and did not show any difference in others [14]. All of these changes were thought to be because of the dry eye of different stages and different severities [15]. In our study, there was no statistical difference in neural innervation between patients and controls. This can be related to the clinical severity and etiological differences of the patients.

The average age was found to be higher in dry eye patients compared to the healthy controls, but no correlation was observed between age and R1 and R2 reflex responses of both eyes. Perhaps if there were more participants, the relationship between age and blink reflex could be examined more clearly.

Blink reflex, which performs electrophysiological structures of facial and trigeminal nerves. Pathologies affecting these nerves and their connections may cause dry eye development. As blinking is very important for the spread of tears to the ocular surface, the healthy functioning of the meibomian glands, and the development of a healthy ocular surface. As the blink reflex test is a noninvasive, fast, and easily applicable method, it can provide useful information for the detection of a pathology related to blinking in dry eye patients. Trigeminal ganglion has been investigated in neurological diseases including Parkinson's, migraine, and idiopathic intracranial hypertension [16-18]. In their study, Samanci et al. found that there is an increase in R2 recovery rates in patients with idiopathic intracranial hypertension in remission. They conclude that this result is because of increased excitability in the trigeminal pathways [17].

There is no study investigating the blink reflex abnormality in dry eye patients in the literature. In our study, no significant difference was found between the patient and control groups in terms of R1 and R2 responses in both eyes. According to these results, we can say that there is no blink reflex pathology in dry eye patients. Regulation of all secretions in the corneal, intraoral, and intranasal region is provided by primary afferent neurons reaching the spinal trigeminal nucleus. Stimulation of the trigeminal sensory ophthalmic branch regulates the release of tears from the lacrimal gland and mucin from the conjunctival mucinous glands via the parasympathetic branch of the facial nerve [19]. Additionally, parasympathetic neurons are also responsible for the secretion of the meibomian glands, which are responsible for lipid secretion. Decreased meibomian gland secretion leads to evaporative dry eye [20]. Abnormalities in these nerves reduce tear secretion. In their study, Shah et al. found that cranial nerve seven palsy leads to eyelid abnormality and meibomian gland dysfunction [21]. In another study, Lee et al. investigated tear meniscus height and clinical assessment of meibomian gland dysfunction in mild facial nerve palsy and found no statistically significant difference between affected and unaffected eyes [22]. Free nerve endings in the cornea transmit corneal sensation via the ophthalmic branch of the trigeminal nerve. The trigeminal nerve is also important for reflex tear release, the blink reflex, and the release of neurotrophic factors that are important for ocular surface health [23,24]. In their study, Altaş et al. found that even if trigeminal neuralgia is unilateral, ocular surface alteration and dry eye are seen in both eyes [25].

The limitation of our study is that the corneal sensitivities of the volunteers included in the study were not measured and compared, and the correlation with the blink reflex was not examined. In future studies, we do not have brainstem fiesta sequence magnetic resonance images (MRI). It could be planned to evaluate central pathologies in more detail. There are many causes of dry eyes. One of them is nerve damage and neural sensitization. The nerve damage can be related to many reasons including peripheral nerve pathologies, trigeminal and facial nerve nucleus pathologies, or pathway abnormalities. If there is a central pathology like pontine pathologies, fiesta MRG would be helpful for the etiology. Fiesta MRI provides clear information in cranial nerve imaging as it distinguishes between cerebrospinal fluid and vascular and nerve tissue using contrast difference. Our study can be considered a preliminary study in this area. Our sample size is small, so the findings cannot be generalized owing to low power. Studies on more volunteers may contribute to the literature on this subject for more statistical power and compare dry eye subgroups.

## Conclusions

The blink reflex is a vital reflex in terms of protecting the ocular surface from external factors. It is controlled by the central nervous system. The afferent innervation of the blink reflex is provided by the supraorbital branches of the trigeminal nerve, and the efferent innervation is provided by the bilateral facial nerve. These two nerves communicate in the central nervous system. In our study, we could not observe any abnormal blink reflex in dry eye disease.
